# Optimization of Thurston’s Core Entropy Algorithm for Polynomials with a Critical Point of Maximal Order

**DOI:** 10.3390/e20090695

**Published:** 2018-09-11

**Authors:** Gamaliel Blé, Domingo González

**Affiliations:** División Académica de Ciencias Básicas, Universidad Juárez Autónoma de Tabasco, Carretera Cunduacán-Jalpa Km 1, Cunduacán Tabasco 86690, Mexico

**Keywords:** core entropy, Thurston’s algorithm, Hubbard tree, external rays

## Abstract

This paper discusses some properties of the topological entropy systems generated by polynomials of degree *d* in their Hubbard tree. An optimization of Thurston’s core entropy algorithm is developed for a family of polynomials of degree *d*.

## 1. Introduction

The topological entropy of a polynomial *P*, denoted by *P* allows us to measure the complexity of the orbits of the dynamical system generated by *P*. This concept has been used to classify the dynamics in different polynomial families, for example, in the case of real one-parameter families of polynomials of degree 2, it has been shown that the entropy behaves monotonically [1,2]. For real cubic maps, it was shown that each locus of constant topological entropy is a connected set [3]. Later, this result was shown for a quartic polynomial family and for real multimodal maps [4,5]. In the complex polynomials family, the entropy is concentrated in the Julia set; it is constant and only depends on the degree of the polynomial family [1,6]. In order to study the dynamics of a polynomial with a finite postcritical set, Douady and Hubbard introduced the Hubbard tree; the theory of admissible Hubbard trees and critical portraits was later studied by Poirier [7]. Afterwards Thurston proposed to study the entropy, restricted to its *Hubbard tree*, of a polynomial with finite postcritical set, which, in this setting, is called the *core entropy*. He showed that the core entropy generalizes the concept defined for an invariant interval in the real case [8]. Furthermore, Thurston proposed an algorithm in order to calculate the core entropy. It is based on a linear transformation *A* (defined in terms of the external arguments of the postcritical set) whose spectral radius coincides with the core entropy [9].

In the case of the quadratic family, Li proved that the core entropy grows through the veins of the Mandelbrot set. Later Tiozzo proved, for the same family, that the core entropy can be extended as a continuous function of the external argument on the boundary of the Mandelbrot set [10,11]. He generalizes this result for polynomials of higher degrees [12].

In this article, we show a simplification of Thurston’s algorithm for a family of polynomials of degree d≥3 with one free critical point and one fixed critical point of maximum multiplicity. We always assume that the free critical point is either periodic or eventually periodic. According to [13], this family is conjugated to

(1) $$P_{a}\left(z\right)=z^{d-1}\left(z+\frac{da}{d-1}\right).$$

The polynomial function $P_{a}\left(z\right)$ has two critical points: zero which is the fixed critical point of maximal multiplicity and −a which is the free critical point. The parameter space of this polynomial family has been studied by Milnor [14,15] in the cubic case, and by Roesch [13], who studied the topological properties of the hyperbolic components in the case of degree d≥3.

To simplify Thurston’s algorithm, we construct a linear transformation $A^{\prime }$ with a definition based on the external arguments of the orbit of the critical point (−a). As we will show, this linear transformation is defined in a space with smaller dimension than the one proposed by Thurston. Consequently, the spectral radius is easier to calculate. Here is the main result of this paper.

**Main** **Theorem.** *Let $P_{a}$ be a postcritically finite polynomial of the family (1). If A denotes the matrix obtained via Thurston’s algorithm, then A and $A^{\prime }$ have equal spectral radii ρ.*

In order to prove the Main Theorem, we use of the concept of external rays, the Thurston algorithm, and some properties of the entropy and non-negative matrices [16,17,18,19,20].

## 2. Thurston’s Algorithm

The algorithm proposed by Thurston allows us to compute the core entropy of a polynom of degree *d*. With the purpose of defining this algorithm, we present some needed concepts which can be found in the work of Gao [9].

### 2.1. The Algorithm of Thurston for Polynomials of Degree d

Let P(z) be a postcritically finite polynomial of degree *d*. Thus, P(z) has exactly d−1 critical points, say, $c_{1},\ldots ,c_{n}$ (counting multiplicities). Each $c_{i}$ is either in the Julia set, $J_{p}$, or is the center of a Fatou component. Furthermore, $J_{p}$ is locally connected [18,21]. The algorithm is based on the analysis of the external rays that land either on the critical points or on the boundaries of Fatou components that contain the critical points.

**Definition** **1.** *We say that an external ray R(θ) supports a bounded Fatou component U if:*

*(1)*  *The ray lands on a point q at the boundary of U.*
*(2)*  *There exists a sector based at q, delimited by R(θ) and the internal ray of U that lands at q, such that the sector does not contain any other external ray that lands on q.*

Given a postcritically finite polynomial of degree *d* and a critical Fatou component *U*, that is, a Fatou component containing a critical point, let $\delta =deg(P{|}_{U})$. We define the set Θ(U) as follows:

(1) If *U* is periodic with orbit
  $$U\to P\left(U\right)\to \ldots \to P^{n}\left(U\right)=U,$$
  we build $\Theta (U^{\prime },z^{\prime },\theta )$ for all $U^{\prime }$ in this orbit simultaneously.

Using the Böttcher coordinates in *U*, we can find z∈∂U with internal argument 0. This *z* is a root of *U*, which depends on the choice of the coordinates. This means that *z* is a periodic point of minimal period on the boundary of U. This choice determines a root for each Fatou component ($P^{k}\left(U\right)$, for k=1,2…,n−1). We call this root a *preferred root of $P^{k}\left(U\right)$*. If $U^{\prime }$ is any component in the cycle and $z^{\prime }$ is its preferred root, consider a ray (R(θ)) which supports $U^{\prime }$ at $z^{\prime }$. Define $\Theta (U^{\prime },z^{\prime },\theta )$ to be the set consisting of $\delta_{U^{\prime }}$ arguments of the support rays for the component $U^{\prime }$ that are the inverse image of P(R(θ)).

(2) If the Fatou component *U* is strictly preperiodic, take *n* as the smallest number for which $P^{n}\left(U\right)$ is a critical Fatou component. Let z∈∂U be such that $P^{n}\left(z\right)=\gamma \left(\alpha \right)$, where γ(α) is the point where R(α) lands on $\partial P^{n}\left(U\right)$ and $\alpha \in \Theta (P^{n}\left(U\right),\gamma \left(\alpha \right),\alpha )$. Consider a ray (R(θ)) that supports component *U* which contains *z*. Define Θ(U,z,θ) as the set of the $\delta_{U}$ arguments of the supporting rays of *U* that, under $P^{n}$, go to R(α).

**Remark** **1.** *For each critical Fatou component U, there exists, at most, a finite number of sets ($\Theta (U^{\prime },z^{\prime },\theta )$), each one dependent on the choice of the root (z) in U and the argument (θ). We can choose any of them and denote it by Θ(U).*

**Definition** **2.** *Let P be a polynomial with finite postcritical set. Let $U_{1},\ldots ,U_{n}$ be the pairwise disjoint critical Fatou components, and let $c_{1},\ldots ,c_{m}$ be the critical points in the Julia set (m+n is the number of different critical points of P). The finite collection of subsets of the circle*

$$\Theta_{P}=\left\{\Theta \left(c_{1}\right),\ldots ,\Theta \left(c_{m}\right),\Theta \left(U_{1}\right),\ldots ,\Theta \left(U_{n}\right)\right\}$$

*is called the critical marking of P, if each of the $\Theta (U_{i})$ is chosen as in Remark 1 and each $\Theta (c_{j})$ consists purely of the angles of the external rays that lands on $c_{j}$.*

Let $\Theta =\left\{\Theta_{1},\Theta_{2}\ldots ,\Theta_{l}\right\}$ be the critical marking of a polynomial *P* of degree *d*. We define the critical and postcritical sets of Θ as

$$crit\left(\Theta \right)=\overset{l}{\underset{k=1}{⋃}}\Theta_{k}\;\;\mathrm{and}\;\;post\left(\Theta \right)=\underset{n\geq 1}{⋃}\tau^{n}crit\left(\Theta \right),$$

respectively, where τ:T→T is the function given by τ(θ)=dθmod1. From the definition of critical marking, it is easy to see that the following holds:

1. Each $\tau \Theta_{i},\;i\in \left\{1,2,\ldots ,l\right\}$, consists of a unique angle.
2. The convex hulls of $\Theta_{i}$ and $\Theta_{j}$ in the unit disk intersect each other in, at most, one point of T, for any i≠j in the set $\left\{1,2,\ldots ,l\right\}$.
3. For each *i*, $\#\Theta_{i}\geq 2$ and $\sum_{i=1}^{l}\left(\#\Theta_{i}-1\right)=d-1$.

Let D be the unit disk endowed with the hyperbolic metric. We identify any point in ∂D with the argument in T. By doing this, each angle in the circle is considered to be mod 1. A leaf is either a point in T or the closure in D of a hyperbolic chord (non-trivial). Indeed, from now on, each time we mention chord or hull in the disk, it will be in the hyperbolic sense. For each set (S⊂T), we denote the convex hull of *S* as a subset of D by hull(S).

A *critical portrait* of degree *d* is a finite collection of finite subsets of the circumference, $\Theta =\left\{\Theta_{1},\Theta_{2}\ldots ,\Theta_{k}\right\}$ satisfying properties **1**, **2**, **3**.

Notice that any critical marking of a postcritically finite polynomial seen in the unit disk is a critical portrait.

**Definition** **3.** *Let $\Theta =\left\{\Theta_{1},\Theta_{2}\ldots ,\Theta_{l}\right\}$ be a critical portrait. Given any two angles x,y∈T that are not necessarily different, and an element Θ of Θ, we say that the chord $\bar{xy}$ crosses the convex hull, hull(Θ) if x,y∉Θ, and $\bar{xy}⋂hull\left(\Theta \right)\neq \emptyset$. In this setting, we also say that x,y are separated by Θ.*

**Definition** **4.** *Given any pair of angles x,y∈T, the separation set relative to Θ is the set $\left\{k_{1},\ldots ,k_{p}\right\}$ where the chord $\bar{xy}$ successively crosses $hull\left(\Theta_{k_{1}}\right),\ldots ,hull\left(\Theta_{k_{p}}\right)$, $\Theta_{k_{j}}\in \Theta$, and no other element of Θ separates the angles x,y. We say that the angles x,y are not separated by Θ if its separation set relative to Θ is empty.*

If $P_{d}$ is a polynomial with finite postcritical set, then each element of its critical portrait Θ is rational and post(Θ) is a finite set. Hence, it is possible to define the finite set *S* consisting of pairs (not ordered) of {x,y} with x≠y∈post(Θ) as long as card(post(Θ))≥2. In the case of post(Θ)={x}, *S* has only one element and in this case, *x* is a fixed point of τ.

Once we have defined the set *S*, the entropy of $P_{d}$ restricted to the Hubbard tree ($H(P_{d})$) is given by the Algorithm 1.

**Algorithm 1** Thurston’s Algorithm
Let V be the vector space over R which has the elements of *S* as a basis.Let A:V→V be the linear transformation defined by the values on the basis *S* of V as follows. For any vector ({x,y}∈S), the image is defined byA({x,y})={τ(x),τ(y)} if x,y are not separated by Θ;$A\left(\left\{x,y\right\}\right)=\sum_{i=0}^{p}A\left(\left\{\theta_{i},\theta_{i+1}\right\}\right)$, where $\theta_{0}=x,\;\theta_{p+1}=y$, and $\theta_{i}\in \Theta_{k_{i}}\in \Theta$, if {x,y} has the separating set $\{k_{1},\ldots k_{p}\}\neq \emptyset$.Let *A* be the matrix associated with the linear transformation A with respect to the basis *S*. As this matrix is non-negative, according to Perron–Frobenius Theorem, the spectral radius ρ of *A* is non-negative [16].

**Theorem** **1** (Gao)**.** *Let $P_{d}$ be a polynomial of degree d with a finite postcritical set, and let Θ be a critical marking for $P_{d}$. If ρ is the spectral radius of the matrix in Thurston’s algorithm, then $h(H\left(P_{d}\right),P_{d})=\log\rho$.*

A full proof of the above Theorem can be found in [9].

One of the advantages of studying the entropy in the critical portrait is the fact that each point of the postcritical set corresponds to an angle in the set post(Θ) in such a way that any arc of $H(P_{d})$ between two vertices can be represented by some pair of angles, although possibly not in a unique way. Intuitively, one can think that the actions of $P_{d}$ in those arcs induce a transformation in the space generated by the pair of angles in the set post(Θ) given by the matrix *A* of Thurston’s algorithm.

### 2.2. Thurston’s Algorithm in the Polynomial Family (1)

Let $P_{a}$ be a polynomial in the family (1). The critical points of $P_{a}$ are 0 and −a. The point 0 is the center of the fixed Fatou component $B_{a}$, and −a is a free critical point. If −a is the center of a Fatou component, then this component will be denoted by $U_{1}$.

We also define the following set of angles

$$\Theta_{0}=\Theta \left(B_{a}\right),\;\;\;\;\;\;\;\;\;\;\;\;\;\;\;\;\;\;\;$$

$$\Theta_{-a}=\left\{\begin{array}{ccc} \Theta (U_{1}) & if & -a\;\mathrm{is}\;\mathrm{the}\;\mathrm{center}\;\mathrm{of}\;a\;\mathrm{Fatou}\;\mathrm{component}\\ \Theta (c_{1}) & if & -a\in J_{a}, \end{array}\;\right.$$

where $\Theta \left(B_{a}\right),\;\Theta \left(U_{1}\right)$ and $\Theta (c_{1})$ are defined as in Section 2.1.

Assume that $P_{a}$ has a finite postcritical set. The collection of angles $\Theta_{P_{a}}=\Theta_{-a}$ is called a restricted critical marking.

Let $\Theta_{P_{a}}$ be the restricted critical marking of $P_{a}$. We define the restricted critical set and the restricted postcritical set as

$$crit\left(\Theta_{P_{a}}\right)=\Theta_{-a}\;\;\mathrm{and}\;\;post\left(\Theta_{P_{a}}\right)=\underset{n\geq 1}{⋃}\tau^{n}crit\left(\Theta_{P_{a}}\right).$$

In the same way as we did before, we identify any point in ∂D with its argument in T. The restricted critical marking of $P_{a}$ viewed in D is denoted by $\Theta_{a}$.

**Lemma** **1.** *If $\Theta_{a}$ is the restricted critical portrait of the polynomial $P_{a}$, then $D\setminus hull(\Theta_{a})$ has 2 connected components with arcs in T of lengths $\frac{1}{d}$ and $\frac{d-1}{d}$, respectively.*

**Proof.**  Since $deg(P{|}_{-a})=2$, $\Theta_{a}$ consists of two elements, the convex hull ($hull(\Theta_{a})$) divides D in two regions. On the other hand, as the elements of $\Theta_{a}$ are obtained as inverse images of the same angle, the arc length between the elements of $\Theta_{a}$ is equal to $\frac{1}{d}$ (c.f. Proposition 2.31 in [13]). This completes the proof. ☐

**Definition** **5.** *Let $\Theta_{a}$ be a restricted critical portrait. Given any two angles x,y∈T (not necessarily different), we say that the chord ($\bar{xy}$) crosses the convex hull ($hull(\Theta_{a})$ of $\Theta_{a}$) if $x,y\notin \Theta_{a}$ and $\bar{xy}⋂hull\left(\Theta_{a}\right)\neq \emptyset$. Under these conditions, we say that x,y are separated by $\Theta_{a}$.*

If $P_{a}$ has a finite postcritical set, then the elements of the restricted critical portrait are rationals, and $post(\Theta_{a})$ is a finite set. We define set $S^{\prime }$ as all pairs (not ordered) of {x,y} with $x\neq y\in post(\Theta_{a})$ as long as $card(post\left(\Theta_{a}\right))\geq 2$. If $post\left(\Theta_{a}\right)=\left\{x\right\}$, then $S^{\prime }$ is the element {x,x}, and *x* is a fixed point of τ. Once we have defined set $S^{\prime }$, the adapted Thurston’s algorithm that is used to approximate the entropy of $P_{a}$ over its Hubbard tree is given by Algorithm 2.

**Algorithm 2** The Adapted Thurston’s Algorithm
Define $V^{\prime }$ as the vector space over R which has the elements of $S^{\prime }$ as a basis.Define the linear transformation $A^{\prime }:V^{\prime }\to V^{\prime }$ by setting the values of $A^{\prime }$ on the basis $S^{\prime }$ of V’ in the following way: For any vector $\left\{x,y\right\}\in S^{\prime }$, the image is defined by$A^{\prime }\left(\left\{x,y\right\}\right)=\left\{\tau \left(x\right),\tau \left(y\right)\right\}$, if x,y are not separated by Θ; and$A^{\prime }\left(\left\{x,y\right\}\right)=\left\{\tau \left(x\right),\tau \left(\theta \right)\right\}+\left\{\tau \left(\theta \right),\tau \left(y\right)\right\}$, $\theta \in \Theta_{a}$ if x,y are separated by Θ.Let $A^{\prime }$ be the matrix associated with the linear transformation $A^{\prime }$ with respect to the basis $S^{\prime }$. This matrix is non-negative, and, according to the Perron–Frobenius Theorem [16], the spectral radius $\rho^{\prime }$ of $A^{\prime }$ is non-negative.

**Theorem** **2.** *Let $P_{a}$ be a finite postcritical polynomial. If A denotes the matrix obtained by Algorithm 1, and $A^{\prime }$ is the matrix generated by Algorithm 2, then A and $A^{\prime }$ have the same spectral radius (ρ).*

**Proof.**  In order to prove the Theorem, we have to consider two cases:

(1) If $-a\in B_{a}$, in this case, the core entropy is zero, and we show that in the restricted algorithm. The spectral radius of matrix $A^{\prime }$ is 1.
(2) If $-a\notin B_{a}$, we show that transformation A can be built without considering the line of separation of the critical point (0).

Let $P_{a}$ be a polynomial of degree *d* with a finite postcritical set. In accordance with Böttcher’s Theorem, a biholomorphism $\phi_{a}$ exists that conjugates $P_{a}$ with the function $z^{d}$ in a neighborhood of infinity. Since $P_{a}$ is postcritically finite, its Julia set is locally connected; hence, $\phi_{a}$ can be extended continuously to $J_{a}$ [21].

Moreover, the dynamics in $B_{a}$ are conjugated to $z^{d-1}$, and the conjugation can be extended continuously to the boundary; hence, a fixed point *p* of $P_{a}$ exists, with an internal angle of 0, that is, in $\partial B_{a}$. The Böttcher coordinate is chosen at infinity in such a way that the external angle of *p* is also 0.

According to the above and the construction of the critical portraits, the set of angles is $\Theta_{0}=\left\{0,\frac{k_{1}}{d},\ldots \frac{k_{d-2}}{d}\right\},$ with $k_{i}\in \left\{1,2,\ldots ,d-1\right\}$. Hence, the critical portrait of $P_{a}$ is given by

$$\Theta =\left\{\left\{0,\frac{k_{1}}{d},\ldots \frac{k_{d-2}}{d}\right\},\Theta_{-a}\right\}\;\;\mathrm{and}\;\;post\left(\Theta \right)=\underset{n\geq 1}{⋃}\tau^{n}\left(\Theta_{-a}\right)⋃\left\{0\right\},$$

where $\Theta_{-a}$ consists of two elements according to Lemma 1.

This shows that for a fixed *d*, the postcritical set varies only in the function of the critical point (−a). On the other hand, the edges of the Hubbard tree are related to the pairs of angles in the critical portrait as follows: the interval of angles with extremes $\left\{\theta_{1},\theta_{2}\right\}$ in the circumference represents a union of edges in the Hubbard tree, and the interval of angles in the circle given by the image of $A(\left\{\theta_{1},\theta_{2}\right\})$ is equivalent to the interval of angles that contains the image under $P_{a}$ of the union of corresponding edges.

**Remark** **2.** *If S denotes the basis of the vector space in Algorithm 1 and the pair $\{\theta_{1},\theta_{2}\}\in S$ is separated with respect to the critical line $\Theta_{c_{i}}$, then the corresponding edge or edges contain the critical point $c_{i}$.*

Case 1: Let $P_{a}$ be a postcritically finite polynomial such that $-a\in B_{a}$. As $P_{a}$ is conjugated to $z^{d-1}$ in $B_{a}$, then the tree of *a* is star shaped with *n* edges. We can label the edges in the Hubbard tree such that the incidence matrix $\tilde{A}=\left(a_{i,j}\right)$ is defined by $a_{i,i+1}=1$ for i=1,…,n−1, $a_{n,j}=1$ for some j∈{i,…,n} and zero otherwise.

The characteristical polynomial of the incidence matrix $\tilde{A}$ is

$${\left(-1\right)}^{n}\lambda^{k-1}\left(\lambda^{n-(k-1)}-1\right),$$

and its spectral radius is 1. Hence $h(P_{a})=0$. On the other hand, Theorem 1 says that the spectral radius of *A* obtained by Thurston’s method is 1.

Due to the fact that the orbit of −a is in $B_{a}$, the restricted critical portrait $\Theta_{a}$ consists of the external angles corresponding to the component $B_{a}$. Hence, pairs ($\left\{\theta_{i},\theta_{j}\right\}$) separated with respect to the critical point (−a) do not exist. Moreover, we can disregard the separation with respect to 0, as in the restricted algorithm. Thus, there is no pair that is separated. Consequently, all pairs $\{\theta_{i},\theta_{j}\}\in S$ have only one image. Furthermore, all rows of matrix $A^{\prime }$ add up to 1; thus, the spectral radius is 1.

Case 2: If $-a\notin B_{a}$, we have the next claim.

**Claim** **1.** *If $\left[v_{1},0\right]$ and $\left[0,v_{2}\right]$ are the two edges of H(a), and $\gamma =\left[v_{1},0\right]⋃\left[0,v_{2}\right]$, then $P_{a}\left(\gamma \right)=\left[P_{a}\left(v_{1}\right),P_{a}\left(v_{2}\right)\right]$.*

**Proof**  If −a is a periodic point, then there are no edges of the forms $\left[v_{1},0\right]$ and $\left[0,v_{2}\right]$ that have the same image. Hence, as 0 is a fixed point, $P_{a}\left(\gamma \right)=\left[P_{a}\left(v_{1}\right),P_{a}\left(v_{2}\right)\right]$.

On the other hand, if −a is preperiodic with $-a\notin B_{a}$, then −a eventually goes to a bifurcation point of $\partial B_{a}$; thus, in this case, there are no edges of the forms $\left[v_{1},0\right]$ and $\left[0,v_{2}\right]$ that go to the same image. ☐

In set *S*, in order to obtain the image of a separated pair ($\left\{\theta_{1},\theta_{2}\right\}$) we can discard the characteristic of being separated with respect to the critical line of $\Theta_{0}$. Thus, for this family of polynomials, if the pair $\left\{\theta_{1},\theta_{2}\right\}$ is separated with respect to Θ, its separation set consists only of one element—the one associated with $\Theta_{-a}$.

Since the postcritical set of $P_{a}$ only depends on the critical point (−a), we can study them if we separate them into the following cases:

(1) If −a is the center of a capture component, then the orbit of $\Theta_{-a}$ eventually contains the zero angle, which is a fixed angle. In this case, the postcritical set of Θ is $\underset{n\geq 1}{⋃}\tau^{n}\left(\Theta_{-a}\right)$. Hence, $A=A^{\prime }$.
(2) If −a eventually goes to $p\in \partial B_{a}$, with a fixed *p*, then the orbit of $\Theta_{-a}$ contains the zero angle; thus, as above, $A=A^{\prime }$.
(3) In any other case, the orbits of $\Theta_{-a}$ and $\Theta_{0}$ are disjoint. Hence, the postcritical set is
  $$Post\left(\Theta \right)=\left\{0,\theta_{1},\ldots \theta_{k}\right\},$$
  where $\theta_{i}=P_{a}^{i}\left(\theta \right)$ with $\theta \in \Theta_{-a}$.

If we write set *S* in such a way that the first *k* elements are of the form $\left\{0,\theta_{i}\right\}$, then *S* can be written as

$$S=\left\{\left\{0,\theta_{1}\right\},\ldots \left\{0,\theta_{k}\right\}\right\}⋃\left\{\{\theta_{i},\theta_{j}\}\;;i\leq j\right\}.$$

Hence, the matrix associated with the transformation A is

$$A=\left(\begin{array}{cc} B & X\\ N & C \end{array}\right),$$

where *B* is the submatrix corresponding to the relations of the images of the pairs of the form $\left\{0,\theta_{i}\right\}$ with themselves, and *C* is the submatrix corresponding to the relations of the images of the pairs $\left\{\theta_{i},\theta_{j}\right\}$ with themselves. The lower submatrix N represents the relations between the images of pairs $\left\{\theta_{i},\theta_{j}\right\}$ with $\left\{0,\theta_{i}\right\}$.

Since we do not consider the separation with respect to the critical line $\Theta_{0}$, and the orbit of $\Theta_{-a}$ does not contain 0, the image of a pair $\left\{\theta_{i},\theta_{j}\right\}$ does not have a component of the form $\left\{0,\theta_{l}\right\}$. Hence, the matrix N is identically 0.

Since *C* is exactly the matrix $A^{\prime }$, to finish the proof of the theorem, it is enough to prove the following claim.

**Claim** **2.** *The spectral radius of matrix B is 1.*

**Proof.** Notice that a pair ($\left\{0,\theta_{i}\right\}$) is fixed under A only when the angle $\theta_{i}$ is fixed. Due to the fact that the only fixed angle of τ is zero and $\theta_{i}\neq 0$, all the elements of the diagonal of *B* are zeros. On the other hand, if $\left\{0,\theta_{i}\right\}$ is not separated, then its image is $\left\{0,\theta_{i+1}\right\}$, and if it is separated, then its image is $\left\{0,\theta_{1}\right\}+\left\{\theta_{1},\theta_{i}\right\}$. In the first case, this generates a 1 over the diagonal of *B*, and in the second case, it generates a 1 on the first column. Thus, *B* has the form

$$B=\left(\begin{array}{cccccc} 0 & 1 & 0 & \ldots , & 0 & 0\\ 0 & 0 & 1 & \ldots , & 0 & 0\\ \vdots & \vdots & \ldots & 1 & \vdots & \vdots \\ 1 & 0 & 0 & \ldots & 0 & 0\\ \vdots & 0 & 0 & \ldots & 0 & 0\\ 1 & 0 & 0 & \ldots & 0 & 0 \end{array}\right)$$

and its spectral radius is then 1. ☐

  ☐

**Figure 1:**
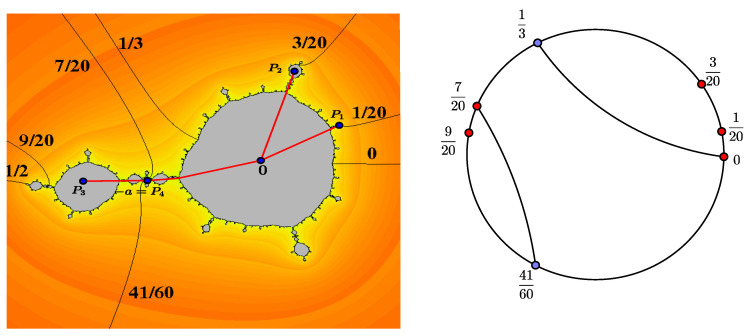
Julia set and critical portrait for d=3 and a=1.07183814+0.1928507i.

**Example** **1.** *Taking d=3 and a=1.07183814+0.1928507i in the polynomial family (1), we have a polynomial with the critical point −a which is periodic with a period of 4. The Julia set is shown in Figure 1.*

*The critical portrait associated with $P_{a}$ is $\Theta =\{\left\{0,\frac{1}{3}\right\},\left\{\frac{7}{20},\frac{41}{60}\right\}\}$ and $post\left(\Theta \right)=\left\{0,\frac{1}{20},\frac{3}{20},\frac{9}{20},\frac{7}{20}\right\}.$ It can be seen in Figure 1.*

*The basis S for the space V is*

$$S=\left\{\left\{0,\frac{1}{20}\right\},\left\{0,\frac{3}{20}\right\},\left\{0,\frac{9}{20}\right\},\left\{0,\frac{7}{20}\right\},\left\{\frac{1}{20},\frac{3}{20}\right\},\left\{\frac{1}{20},\frac{9}{20}\right\},\left\{\frac{1}{20},\frac{7}{20}\right\},\left\{\frac{3}{20},\frac{9}{20}\right\},\left\{\frac{3}{20},\frac{7}{20}\right\},\left\{\frac{9}{20},\frac{7}{20}\right\}\right\}.$$

*By applying the linear transformation A to the elements of the basis, we obtain*

$$\begin{array}{ll} \left\{0,\frac{1}{20}\right\}\mapsto \left\{0,\frac{3}{20}\right\} & \;\;\left\{\frac{1}{20},\frac{9}{20}\right\}\mapsto \left\{0,\frac{3}{20}\right\}+\left\{0,\frac{1}{20}\right\}+\left\{\frac{1}{20},\frac{7}{20}\right\}\\ \left\{0,\frac{3}{20}\right\}\mapsto \left\{0,\frac{9}{20}\right\} & \;\;\left\{\frac{1}{20},\frac{7}{20}\right\}\mapsto \left\{0,\frac{3}{20}\right\}+\left\{0,\frac{1}{20}\right\}\\ \left\{0,\frac{9}{20}\right\}\mapsto \left\{0,\frac{1}{20}\right\}+\left\{\frac{1}{20},\frac{7}{20}\right\} & \;\;\left\{\frac{3}{20},\frac{9}{20}\right\}\mapsto \left\{0,\frac{9}{20}\right\}+\left\{0,\frac{1}{20}\right\}+\left\{\frac{1}{20},\frac{7}{20}\right\}\\ \left\{0,\frac{7}{20}\right\}\mapsto \left\{0,\frac{1}{20}\right\} & \;\;\left\{\frac{3}{20},\frac{7}{20}\right\}\mapsto \left\{0,\frac{9}{20}\right\}+\left\{0,\frac{1}{20}\right\}\\ \left\{\frac{1}{20},\frac{3}{20}\right\}\mapsto \left\{\frac{3}{20},\frac{9}{20}\right\} & \;\;\left\{\frac{9}{20},\frac{7}{20}\right\}\mapsto \left\{\frac{1}{20},\frac{7}{20}\right\}. \end{array}$$

*Hence, the matrix associated with the linear transformation A is*

$$A=\left(\begin{array}{cccccccccc} 0 & 1 & 0 & 0 & 0 & 0 & 0 & 0 & 0 & 0\\ 0 & 0 & 1 & 0 & 0 & 0 & 0 & 0 & 0 & 0\\ 1 & 0 & 0 & 0 & 0 & 0 & 1 & 0 & 0 & 0\\ 1 & 0 & 0 & 0 & 0 & 0 & 0 & 0 & 0 & 0\\ 0 & 0 & 0 & 0 & 0 & 0 & 0 & 1 & 0 & 0\\ 1 & 1 & 0 & 0 & 0 & 0 & 1 & 0 & 0 & 0\\ 1 & 1 & 0 & 0 & 0 & 0 & 0 & 0 & 0 & 0\\ 1 & 0 & 1 & 0 & 0 & 0 & 1 & 0 & 0 & 0\\ 1 & 0 & 1 & 0 & 0 & 0 & 0 & 0 & 0 & 0\\ 0 & 0 & 0 & 0 & 0 & 0 & 1 & 0 & 0 & 0 \end{array}\right),$$

*and its spectral radius is 1.3953. In accordnce with Theorem 1, we conclude that the entropy of $P_{a}$, restricted to its Hubbard tree, is log1.3953.*

**Figure 2:**
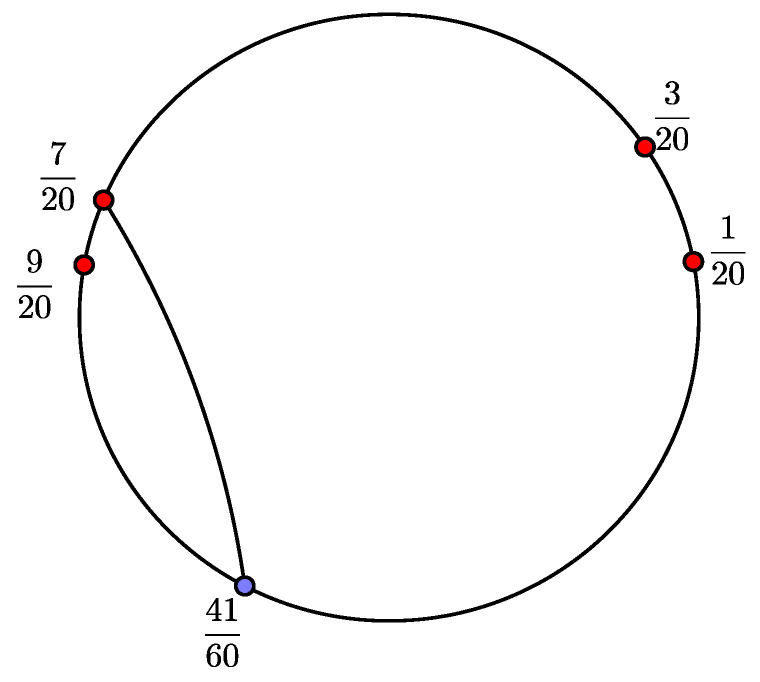
Restricted critical portrait for d=3 and a=1.07183814+0.1928507i.

*On the other hand, the restricted critical portrait associated with $P_{a}$ is $\Theta =\{\left\{\frac{7}{20},\frac{41}{60}\right\}\}$, and $post\left(\Theta \right)=\left\{\frac{1}{20},\frac{3}{20},\frac{9}{20},\frac{7}{20}\right\}$. It can be seen in Figure 2.*

*The basis S of the space V is given by*

$$S=\left\{\left\{\frac{1}{20},\frac{3}{20}\right\},\left\{\frac{1}{20},\frac{9}{20}\right\},\left\{\frac{1}{20},\frac{7}{20}\right\},\left\{\frac{3}{20},\frac{9}{20}\right\},\left\{\frac{3}{20},\frac{7}{20}\right\},\left\{\frac{9}{20},\frac{7}{20}\right\}\right\}.$$

*The transformation* $A^{\prime }$ *on the basis S is*

$$\begin{array}{ll} \left\{\frac{1}{20},\frac{3}{20}\right\}\mapsto \left\{\frac{3}{20},\frac{9}{20}\right\} & \;\;\left\{\frac{3}{20},\frac{9}{20}\right\}\mapsto \left\{\frac{1}{20},\frac{9}{20}\right\}+\left\{\frac{1}{20},\frac{7}{20}\right\}\\ \left\{\frac{1}{20},\frac{9}{20}\right\}\mapsto \left\{\frac{1}{20},\frac{3}{20}\right\}+\left\{\frac{1}{20},\frac{7}{20}\right\} & \;\;\left\{\frac{3}{20},\frac{7}{20}\right\}\mapsto \left\{\frac{1}{20},\frac{9}{20}\right\}\\ \left\{\{\frac{1}{20},\frac{7}{20}\right\}\mapsto \left\{\frac{1}{20},\frac{3}{20}\right\} & \;\;\left\{\frac{9}{20},\frac{7}{20}\right\}\mapsto \left\{\frac{1}{20},\frac{7}{20}\right\} \end{array}$$

*The associated matrix is*

$$A=\left(\begin{array}{cccccc} 0 & 0 & 0 & 1 & 0 & 0\\ 1 & 0 & 1 & 0 & 0 & 0\\ 1 & 0 & 0 & 0 & 0 & 0\\ 0 & 1 & 1 & 0 & 0 & 0\\ 0 & 1 & 0 & 0 & 0 & 0\\ 0 & 0 & 1 & 0 & 0 & 0 \end{array}\right),$$

*with a spectral radius of 1.3953.*

**Figure 3:**
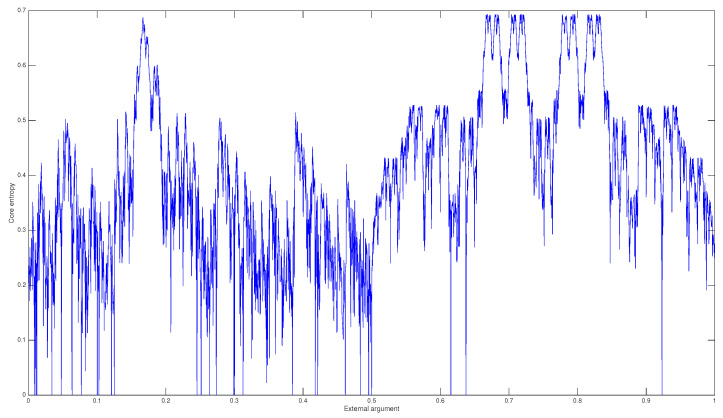
Core entropy for d=3.

As the above example shows, the Thurston restricted algorithm allows us to reduce the dimensions of the matrix as well as the cardinality of the orbit of −a. Furthermore, the sum of the elements of any row of $A^{\prime }$ is, at most, 2, while the sum of the elements of a row in *A* can be greater than 2. Figure 3 shows the core entropy as a function of the external argument for d=3.
